# Compromised RNA polymerase III complex assembly leads to local alterations of intergenic RNA polymerase II transcription in *Saccharomyces cerevisiae*

**DOI:** 10.1186/s12915-014-0089-x

**Published:** 2014-10-28

**Authors:** Qing Wang, Chance M Nowak, Asawari Korde, Dong-Ha Oh, Maheshi Dassanayake, David Donze

**Affiliations:** Department of Biological Sciences, Louisiana State University, Baton Rouge, LA 70803 USA

**Keywords:** RNA polymerase III, Extra-transcriptional effects, Transcriptome, TFIIIC, *ETC* sites, 5′-UTR

## Abstract

**Background:**

Assembled RNA polymerase III (Pol III) complexes exert local effects on chromatin processes, including influencing transcription of neighboring RNA polymerase II (Pol II) transcribed genes. These properties have been designated as ‘extra-transcriptional’ effects of the Pol III complex. Previous coding sequence microarray studies using Pol III factor mutants to determine global effects of Pol III complex assembly on Pol II promoter activity revealed only modest effects that did not correlate with the proximity of Pol III complex binding sites.

**Results:**

Given our recent results demonstrating that tDNAs block progression of intergenic Pol II transcription, we hypothesized that extra-transcriptional effects within intergenic regions were not identified in the microarray study. To reconsider global impacts of Pol III complex binding, we used RNA sequencing to compare transcriptomes of wild type versus Pol III transcription factor TFIIIC depleted mutants. The results reveal altered intergenic Pol II transcription near TFIIIC binding sites in the mutant strains, where we observe readthrough of upstream transcripts that normally terminate near these sites, 5′- and 3′-extended transcripts, and de-repression of adjacent genes and intergenic regions.

**Conclusions:**

The results suggest that effects of assembled Pol III complexes on transcription of neighboring Pol II promoters are of greater magnitude than previously appreciated, that such effects influence expression of adjacent genes at transcriptional start site and translational levels, and may explain a function of the conserved *ETC* sites in yeast. The results may also be relevant to synthetic biology efforts to design a minimal yeast genome.

**Electronic supplementary material:**

The online version of this article (doi:10.1186/s12915-014-0089-x) contains supplementary material, which is available to authorized users.

## Background

In eukaryotes, there are three major types of RNA polymerase designated as Pol I, II, and III (with additional polymerase complexes in plants), which function to transcribe the vast array of RNA species that contribute to the highly complex and heterogeneous eukaryotic transcriptome. Pol I transcribes the majority of ribosomal RNAs, and Pol II is mainly dedicated to protein coding genes. RNA polymerase III (Pol III) transcribes genes encoding small non-translated RNAs, which in the budding yeast *Saccharomyces cerevisiae* includes transfer RNAs (tRNAs), 5S ribosomal RNA (5S rRNA), 7SL RNA, U6 spliceosome RNA, snR52 small nucleolar RNA as well as the RNA component of RNaseP [1-3]. These diverse genes contain three types of promoter element arrangements. The tRNA genes (tDNAs) utilize what is referred to as a type 2 internal promoter, and the transcription factor binding sites within these genes are referred to as internal control regions (ICRs). Type 2 promoters contain conserved A-box and B-box ICR elements separated by a variable distance. These sequences serve as binding sites for the multi-subunit transcription factor TFIIIC [4-6].

In yeast, Pol III transcription of tDNAs requires binding of three multimeric protein complexes – TFIIIC (six polypeptides), TFIIIB (three polypeptides) and Pol III enzyme (seventeen polypeptides). Pol III complex assembly at tDNAs is initiated by the binding of TFIIIC, which then recruits TFIIIB followed by Pol III [4]. The binding affinity of TFIIIC is primarily determined by B-box interactions, and mutation of an invariant cytosine residue in the B-box consensus sequence GWTCRANNC severely diminishes TFIIIC binding affinity and subsequent transcriptional activity of the mutated tDNA [3,7,8]. In addition to Pol III transcribed genes, TFIIIC complexes appear to be bound to other chromosomal locations in the absence of TFIIIB and Pol III [9,10], and in *S. cerevisiae* such locations have been referred to as extra-TFIIIC (*ETC*) sites [11].

In addition to promoting small RNA transcription, Pol III complexes assembled on eukaryotic chromosomes are responsible for what has been termed ‘product independent’ or ‘extra-transcriptional’ functions [5,12]. Characterized extra-transcriptional effects of Pol III complexes, mainly studied in *S. cerevisiae*, include targeting yeast Ty retroelement integration [13-15], phasing of local nucleosome positioning [16-18] and pausing of DNA polymerase progression as replication forks encounter tDNAs [19,20]. Additional effects include inhibition of transcription from nearby Pol II promoters, referred to as tRNA gene mediated (tgm) silencing [21] or position effects [22], and also include both barrier and insulator types of chromatin boundary activities [23,24]. Sequences that recruit the TFIIIC complex have also been shown to have chromatin boundary-like activities in other eukaryotes [25-29]. Most recently, our lab demonstrated in *S. cerevisiae* that a tDNA acts as a roadblock to cryptic intergenic transcription [30]. This latest study showed that either mutation of the tDNA upstream of *ATG31* or global impairment of Pol III complex formation allowed readthrough of the *SUT467* non-coding intergenic transcript through the tDNA region. Readthrough at this site resulted in the production of extended *SUT467-ATG31* hybrid transcripts. These transcripts are defective for translation of Atg31p due to the extended 5′-untranslated region (5′-UTR), which results in reduced fitness under nitrogen starvation conditions due to under-expression of this critical autophagy protein.

A previous study was performed to assess genome-wide extra-transcriptional effects of assembled Pol III complexes on Pol II transcribed genes by comparing coding sequence microarray expression levels of wild type versus a variety of Pol III defective mutant yeast strains. Mutant subunits of TFIIIC, TFIIIB or Pol III resulted in minimal effects on expression levels of genes adjacent to tDNAs and *ETC* sites, and most of the differences observed were due to secondary effects mediated by activation of Gcn4p transcription factor activity in response to reduced initiator tRNA^Met^ levels [31].

Since we observed changes in *intergenic* transcription upstream of *ATG31* upon mutation of the adjacent tDNA and in mutants under-expressing the TFIIIC subunit Tfc6p, we revisited the genome-wide analysis of Pol III complex mediated extra-transcriptional effects using high-throughput RNA-Sequencing (RNA-Seq). We reasoned that RNA-Seq would identify differences in intergenic transcription that were missed in the previous microarray analysis. The results presented here comparing wild type yeast to Tfc6p under-expressing mutants recapitulate the Gcn4p mediated effects from the Conesa *et al*. [31] study. Additionally, numerous alterations in intergenic transcription in close proximity to tDNAs and other Pol III complex binding sites are observed in *tfc6* mutants. Analysis of loci adjacent to Pol III complex binding sites that were significantly altered in the *tfc6* mutants reveal both 5′- and 3′-extended transcripts, along with increased intergenic transcription and the de-repression of a meiosis-specific transcript. Extension of the 5′-end of transcripts compromises coding sequence translation as expected. The results are discussed in terms of the origins and impact of these altered RNAs, the role of the Pol III complex as a type of boundary element, and how compromising association of particular chromatin binding complexes can have unforeseen global impacts on both the transcriptome and the proteome.

## Results

### Mapping and analysis of RNA-Seq reads in wild type and *tfc6*-under-expressing strains

To assess the genomic impact of RNA Pol III complex assembly on neighboring Pol II genes, we performed high-throughput RNA-Seq of ribosomal RNA depleted samples from two types of yeast strains: wild type and Tfc6p-under-expressing strains. In this study, we used strains DDY4300 and DDY4301 (referred to from here as *tfc6* mutants). These previously characterized strains contain a *TFC6* promoter mutation that leads to under-expression of *TFC6* mRNA and a slow growth phenotype [32], but were not assessed for Tfc6 protein levels. As we learned in the course of this study, the level of Tfc6p protein expression in these mutants was much lower than we had previously assumed based on the approximately twofold reduction in *TFC6* mRNA levels (see below). Total RNA was extracted from two independently isolated wild type and two *tfc6* mutant strains at mid-log phase growth in rich media (A_600_ 1.0) and processed for RNA-Seq (see Materials and Methods). In total, 196,295,402 strand-specific 100 base reads were generated for the four RNA samples using Illumina HiSeq2000 technology. Across all four samples, the number of sequenced reads ranged from approximately 38 to approximately 55 million, and approximately 95% to 97% of the reads from each sample were uniquely mapped to the yeast genome. Of these sequence reads, 5.3 +/- 0.1% were mapped to annotated open reading frame (ORF) antisense strands, which is consistent with past studies demonstrating antisense transcripts in yeast [33-35]. Correlation co-efficiency was calculated between the two biological replicates in each condition (r =0.96 in wild type; r =0.99 in *tfc6* mutants), which indicated a high correlation between our biological replicates.

### Detection of differentially expressed (DE) genes and DE intergenic Pol II transcripts between wild type and *tfc6* mutants

As described above, a recent study from our lab found that the Pol III complex bound at a tDNA functions to block cryptic intergenic transcription [30]. This was demonstrated by compromising Pol III complex assembly at the particular tDNA, which allowed transcribing intergenic Pol II to read through the tDNA sequence into the downstream gene, creating 5′-extended hybrid RNA molecules. By globally weakening TFIIIC complex assembly at all chromosomal locations, we anticipated observing additional intergenic alterations of Pol II transcription. RNA-Seq analysis revealed numerous such intergenic changes adjacent to Pol III factor binding sites in the *tfc6* mutants. A custom pipeline was developed to count RNA-Seq reads mapped to the annotated gene space (including both ORFs and non-coding RNA genes) and to intergenic regions (see Materials and Methods). The mapped read counts for each sample are presented in Additional file 1: Table S1. These counts were then subjected to DESeq analysis to identify gene ORFs or intergenic regions that are DE between the wild type and *tfc6*-under-expressing mutants (see Additional file 1: Figure S1).

**Figure 1:**
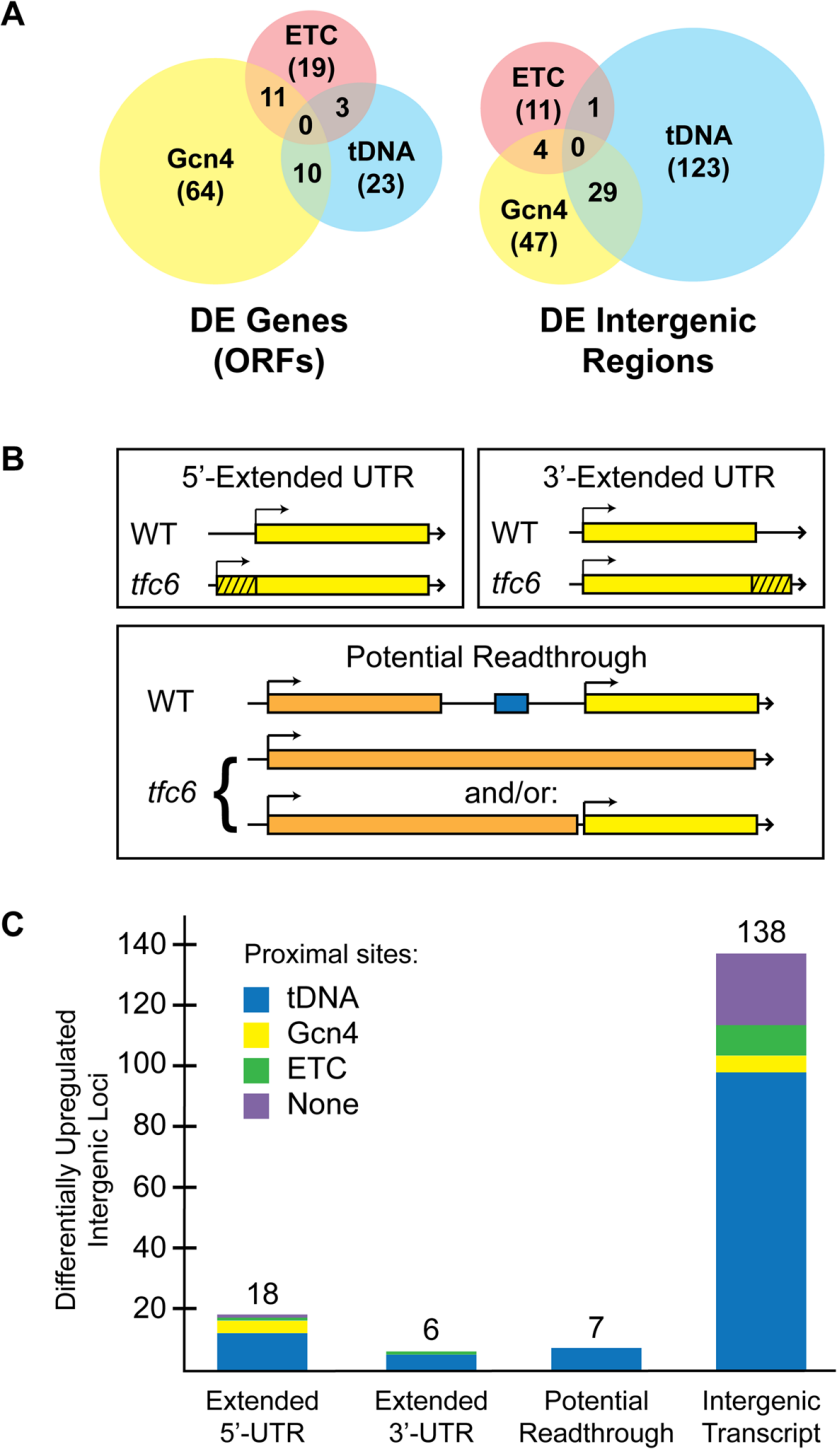
**Differentially expressed (DE) genes and intergenic regions in Tfc6p under-expressing mutants. A)** Distribution and overlap of DE open reading frame sequences versus intergenic regions. Locations adjacent to tDNAs are based on known annotations, and those adjacent to potential Gcn4p sites and *ETC* sites are based both on annotations and on the chromatin immunoprecipitation data available on the *Saccharomyces* Genome Database (references [36] and [37]). Total numbers for each group are in parentheses. **B)** Schematic of types of mis-expression observed in *tfc6* mutants at DE intergenic regions that are contiguous with normal transcripts. This does not include appearance or up-regulation of cryptic transcripts identified in the DESeq analysis. The tDNA sequence is depicted as the blue box. **C)** Categorization of up-regulated DE intergenic transcripts in the *tfc6* mutants. Total numbers of loci in each category are listed above the bars.

Using an adjusted cutoff value of padj <0.05, we observed 99 significantly DE coding regions (DE genes described above) and 173 intergenic regions showing differential expression (DE intergenic regions in Figure 1A). A total of 169 DE intergenic regions were de-repressed in *tfc6* mutants, and most appear to be due to inadvertent cryptic Pol II transcription originating near compromised tDNAs. Additional file 1: Table S2 categorizes DE transcripts in both protein-coding and intergenic regions, based on their proximities to potential Gcn4p and TFIIIC binding sites. Some of these cryptic transcripts appear to arise from bidirectional transcription from neighboring promoters that occurs when the Pol III complex is compromised (see Discussion below). The DESeq results are provided in Additional file 2: Table S3 and Additional file 3: Table S4, and include descriptions for DE intergenic transcripts that are adjacent to potential Pol III complex-binding sites, and show significantly altered (padj <0.05) expression in the *tfc6* mutants. The Conesa *et al*. [31] study showed that most up-regulation of coding sequences was due to the induction of Gcn4 protein production, and the majority of mis-expressed Pol II transcripts did not correlate with the proximity to tDNAs or other TFIIIC associated sites. Here we observed a similar Gcn4p regulated pattern for coding regions, as most of the top 24 up-regulated genes identified in the Conesa *et al*. [31] microarray analysis were also increased in our *tfc6* mutants analyzed by RNA-Seq (see Additional file 1: Table S5), with two explainable exceptions (see Additional file 1: Table S5 legend). However, in contrast to the microarray study, a clear association of intergenic transcriptional mis-regulation with proximity to Pol III factor binding sites was evident in the *tfc6* strains as described below.

The data represented in Additional file 1: Table S2 and Figure 1A show that 39 out of 99 DE ORFs have either a tDNA or *ETC* site nearby (between the DE ORF and the next annotated ORF); however, many of these TFIIIC binding sites may be too far away to be responsible for the observed up-regulation. *ETC* sites include those verified in previous studies [9-11] and potential sites that appear to associate with the TFIIIC complex according to genome-wide chromatin immunoprecipitation data available through the *Saccharomyces* Genome Database [36,37]. Within intergenic regions, we observed several types of transcriptional mis-regulation in addition to simple up-regulation of intergenic transcripts, including 5′-extended, 3′-extended, and potential readthrough transcripts (schematically depicted in Figure 1B). For DE intergenic regions, 133/173 (approximately 77%) have a tDNA or *ETC* site in close proximity, most within approximately 500 base pairs. Among these differentially expressed regions in *tfc6* mutants, we found 18 that are consistent with 5′-UTR extensions, 6 are apparent 3′-UTR extensions, and 138 are up-regulated intergenic transcripts (including some retroelement and long terminal repeat (LTR) regions, Figure 1C). Seven DE intergenic regions appear to be readthrough transcripts from upstream promoters similar to what we observed by mutating the tDNA upstream of *ATG31* [30]. In order to determine whether these effects may be directly due to compromised binding of Pol III complexes, we manually inspected each region. We found that all seven readthrough transcripts were associated with an overlapping tDNA. Of the 18 5′-UTR extensions, 16 are adjacent to tDNAs or *ETC* sites. Five out of six 3′-UTR extensions had tDNAs at the readthrough sites, and 105 out of 138 (76%) intergenic de-repressed regions had either a tDNA or *ETC* site in close proximity. These results indicated a high degree of correlation between the presence of Pol III complexes and mis-expression of intergenic regions when Tfc6p was under-expressed.

We were also able to verify altered Pol II transcription in the *tfc6* mutants at regions known previously to be affected by specific tDNA mutations. Our recent study of the *ATG31-tV(UAC)D-SES1* locus identified the *SUT467-ATG31* readthrough transcript [30] when the intervening tDNA gene was mutated. We detected this locus as an up-regulated readthrough transcript by DESeq analysis (see Additional file 3: Table S4) and by manual inspection of the Integrative Genomics Viewer (IGV, see below) transcription profile (see Additional file 1: Figure S2). DESeq analysis also verified increased *CBT1* [24,38] and decreased *GIT1* mRNA levels [23], consistent with previous studies where the neighboring tDNAs were specifically mutated (see Additional file 3: Table S4). Given these results, we conclude that this transcriptome analysis of Tfc6p under-expressing mutants accurately identifies global extra-transcriptional impacts of chromatin bound Pol III complexes.

### *TFC6* promoter mutants are impaired at both transcriptional and translational levels

**Figure 2:**
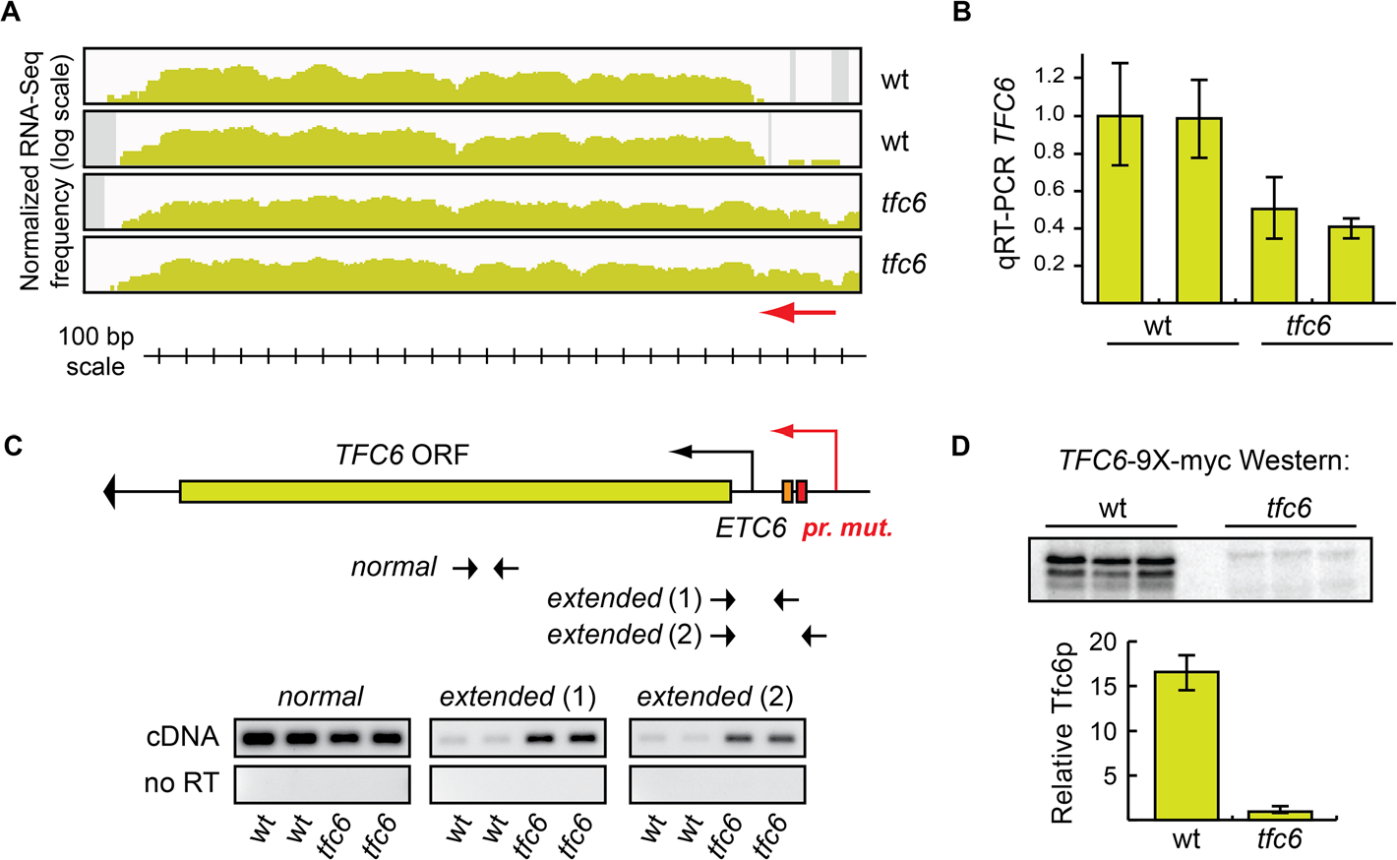
**Tfc6 protein expression is reduced due to both transcriptional and translation defects in the promoter mutant strain. A)** Integrative Genomics Viewer (IGV) transcription profile of the *TFC6* gene region in wild type (DDY3 and DDY3630) and *tfc6* mutant strains (DDY4300 and DDY4301, full strain genotypes are listed in Additional file 4: Table S7). All IGV profiles here and in subsequent figures are displayed on log scale, with the Y-axis representing normalized RNA-Seq frequency, and the X-axis the chromosomal region. The red arrow indicates the range of the extended 5′-UTR region in the mutants. **B)** Relative levels of *TFC6* mRNA in each strain determined by qRT-PCR using primers within the coding sequence, and the same RNA samples used in the RNA-Seq library preparation. **C)** Schematic of the *TFC6* gene and promoter region, showing the relative locations of the *ETC6* site and the promoter mutation. The figure is drawn to scale and aligned with the IGV profile and scale bar in panel **A)** above. The large arrowhead at the end of the gene indicates the direction of transcription. RT-PCR was performed on the same RNA samples as in **B)** using primers specific to the normal (within the open reading frame) and 5′-UTR extended transcripts. Extended transcripts were highly enriched in the *tfc6* mutant strains. **D)** Three independently isolated *TFC6*-9 × -myc tagged strains were constructed from wild type and *tfc6* promoter mutants, and the relative levels of Tfc6 protein produced in each strain were determined by Western blotting. Quantitation of the Western blot signals showed an approximate 17-fold reduction in Tfc6p in the mutants.

*TFC6* (*YDR362C*) encodes the Tfc6 protein, a subunit of the RNA polymerase III transcription initiation factor complex TFIIIC that cooperates with the Tfc3p subunit to bind to the B-box consensus of Pol III internal promoters [39]. As described above, the *tfc6* promoter mutants used here were previously shown to under-express *TFC6* mRNA [32], but protein levels were not determined. Interestingly, inspection of the RNA-Seq transcript profile at the *TFC6* locus in these promoter mutant strains (Figure 2A) using IGV software [40,41] suggested that in addition to the modest decrease in mRNA levels in the mutants, a fraction of the *TFC6* transcripts appears to initiate farther upstream, leading to an extended 5′-UTR (extension depicted as the red arrow in Figure 2A). To confirm the decrease in mRNA levels, we performed quantitative reverse transcript PCR (qRT-PCR) on the same RNA preparations used for RNA-Seq. In *tfc6* promoter mutants, *TFC6* mRNA levels were decreased to 40% to 50% relative to wild type (Figure 2B), consistent with our previous estimate of approximately 50% determined by Northern blot quantitation [32]. DESeq analysis of the RNA-Seq data indicated a similar reduction in *TFC6* mRNA levels in the mutants to approximately 60% of wild type (see Additional file 1: Table S3).

This apparent 5′-UTR extension of the *TFC6* mRNA was verified by RT-PCR analysis using a coding sequence primer pair and two different primer pairs to amplify sequences upstream of the normal *TFC6* transcription start sites. The results in Figure 2C demonstrate that 5′-extended transcripts were enriched in *tfc6* mutants compared to wild type as assayed by both primer pairs. We speculated that this increase in the length of the 5′-UTR in a fraction of *TFC6* mRNA molecules might impair translation of those mRNAs, and reduce Tfc6 protein levels more than expected based on the approximately 50% reduction in mRNA levels, as new AUG and stop codons would be present upstream of the annotated *TFC6* AUG codon. To determine whether translation of Tfc6p was affected in *tfc6* promoter mutants, we integrated nine copies of the myc epitope tag coding sequence onto the 3′-end of the *TFC6* gene in wild type and *tfc6* mutants to create carboxy-terminal 9 × -myc epitope tagged strains. Western blot analysis was performed on three independently isolated wild type and *tfc6* promoter mutant *TFC6*-9 × -myc strains using anti-myc antibody, and images were quantified using Bio-Rad ImageLab software. Figure 2D shows that Tfc6 protein levels were reduced by approximately 17-fold in the mutant strains compared to wild type strains. Given this drastic reduction in Tfc6p levels, we conclude that global Pol III complex assembly is more severely compromised in these promoter mutant strains than was previously appreciated based on mRNA levels, and that these are ideal mutants to assess genome-wide extra-transcriptional effects of Pol III complex formation.

### Confirmation of transcriptome analysis interpretations by site-specific B-box mutations

#### 5′ UTR Extensions

**Figure 3:**
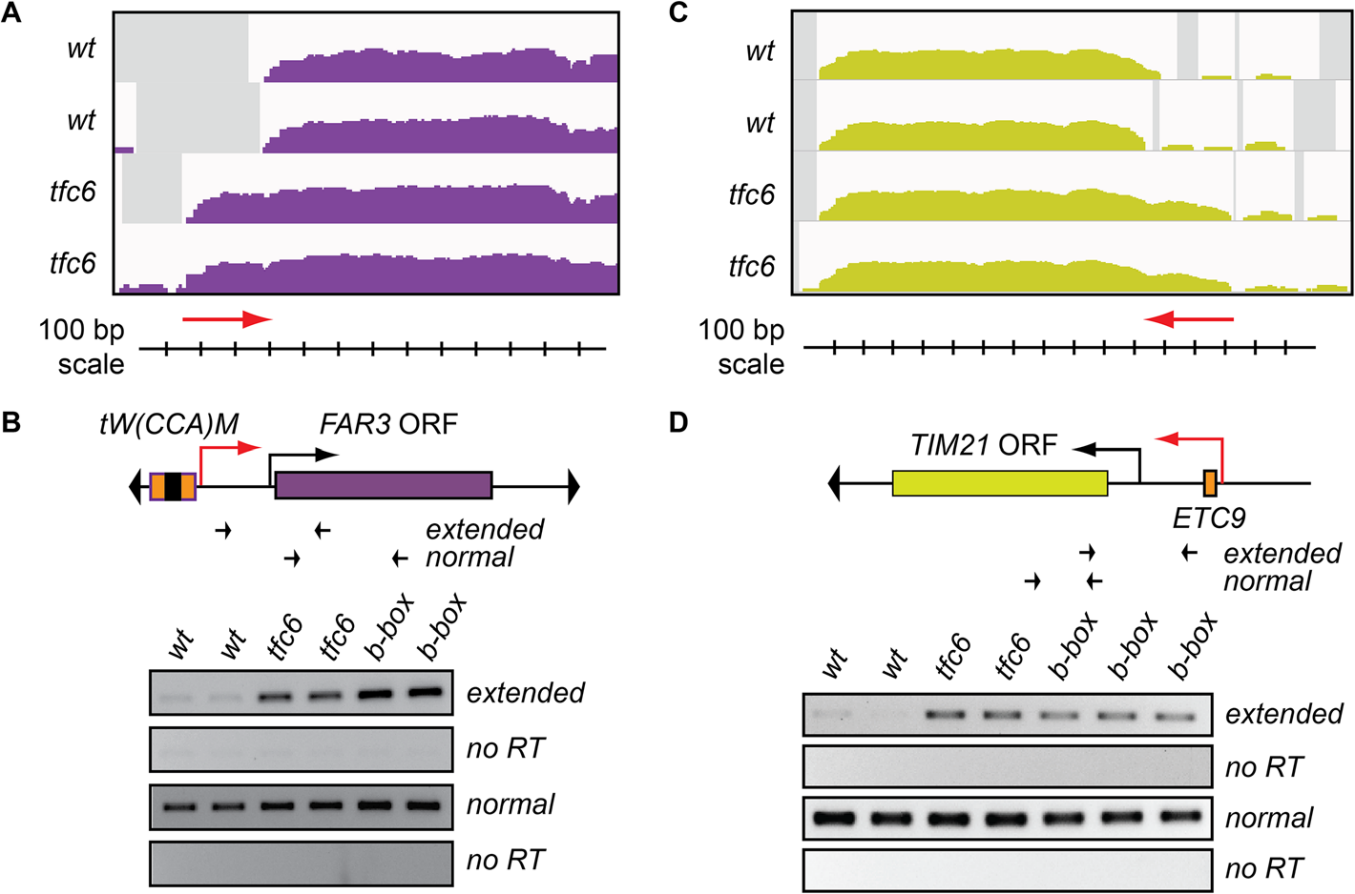
**RT-PCR confirmation of extended 5′-UTRs when adjacent Pol III binding sites are compromised. A)** IGV profiles of RNA-Seq reads at the *FAR3* locus in wild type and *tfc6* mutant strains. Red arrow indicates extended 5′-UTR. **B)** Schematic diagram of the *FAR3* locus showing relative location of upstream tDNA and location of primers to detect extended transcripts. Black promoter arrow indicates normal TSS, red promoter arrow represents the putative upstream TSS in the mutant backgrounds. RT-PCR results show enrichment of extended transcripts in both *tfc6* and specific tDNA B-box mutants relative to wild type, while no significant differences in amplification were observed using primers within the open reading frame. **C)** and **D)** Similar analysis at the *TIM21* locus, which is downstream of the tRNA pseudogene *ETC9*. Wild-type and *tfc6* mutants are the same as in Figure 2, B-box mutants are strains DDY5118 and DDY5120 for the *FAR3* locus and DDY4093-4095 for the *TIM21* locus. IGV, integrative genomics viewer; TSS, transcription start site.

Figure 3 depicts two example loci adjacent to assembled Pol III complexes that appeared to have extended 5′-UTRs in the *tfc6* mutant strains: *FAR3* (*YMR052W*) and *TIM21* (*YGR033C*). *FAR3* is located on *S. cerevisiae* chrXIII, with a tDNA (*tW(CAA)M*) approximately 200 base pairs upstream. Inspection of IGV profiles revealed that *FAR3* had an apparent extended 5′-UTR in the *tfc6* mutants (Figure 3A, red arrow), which is supported by DESeq analysis that showed a statistically significant (padj <0.05) 21-fold increase of reads in this region. In order to confirm this as a 5′-UTR extension, we performed RT-PCR using RNA extracted from the wild type and *tfc6* mutants. Included in the analysis were two independent strains engineered to contain mutations in the B-box of *tW(CAA)M* to disrupt Pol III complex assembly specifically at the adjacent tDNA locus. To verify that these alterations in the IGV transcript profile were due to true extensions and not separate RNA species, RT-PCR primers were designed to amplify cDNA produced from the normal *FAR3* mRNA as controls (within the coding sequence), along with a second set specific for cDNAs generated from 5′-extended mRNAs. Figure 3B shows RT-PCR results with these two sets of primers. Wild type strains exhibited weak amplification of the extended cDNA, while *tfc6* mutants and B-box mutants showed stronger signals, consistent with the presence of 5′-UTR extensions in strains where adjacent Pol III complex assembly was either globally or site-specifically impaired. Control primers within the coding sequence showed similar amplification in all samples.

As a second verification of an extended 5′-UTR, we performed a similar analysis on the *TIM21* locus adjacent to an *ETC*-like site. *TIM21* is located on chrVII, approximately 330 base pairs downstream of the tRNA pseudogene *ETC9*, which has been shown to recruit mainly TFIIIC and TFIIIB, but not Pol III [42]. DESeq analysis showed a significant 4.8 fold enrichment of RNA-Seq reads upstream of the normal *TIM21* 5′-UTR in the *tfc6* mutants, which is evident by inspection of the IGV profile (Figure 3C, red arrow). To verify the observed difference in the profile as a 5′-extension, we performed RT-PCR as above, including three independently isolated *etc9* B-box mutant strains. The RT-PCR results in Figure 3D are again consistent with our interpretation of the IGV transcription profiles, as 5′-extended *TIM21* transcripts were detected at higher levels in *tfc6* and *etc9 B-box* mutant strains compared to wild type. In summary, IGV, DEseq and RT-PCR results were all consistent with the existence of 5′-UTR extensions on both *FAR3* and *TIM21* mRNAs when Pol III complex assembly is inhibited at the adjacent tDNA or *ETC* sites. Additional file 1: Table S6 lists the most significantly affected genes with potential 5′-extensions and other observed alterations of intergenic transcription.

#### 3′-UTR Extensions

**Figure 4:**
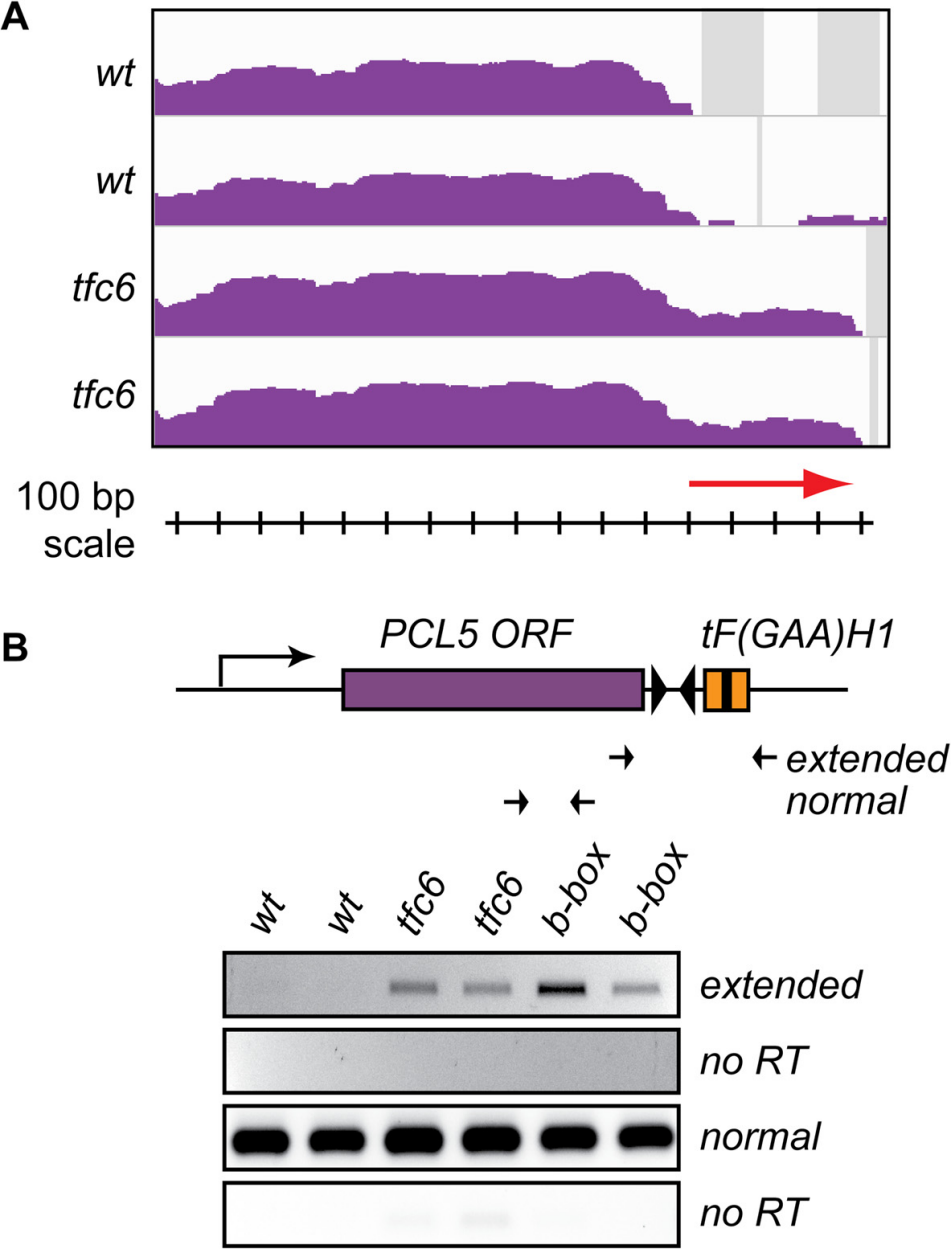
**RT-PCR confirmation of extended 3′-UTR at** ***PCL5*** **. A)** IGV profiles for the *PCL5* locus, with red arrow denoting the 3′-UTR extension. **B)** Schematic of locus as in Figure 3, showing the downstream tDNA, with RT-PCR primers designed to detect the 3′-extension. Results again confirm extended transcripts in *tfc6* and B-box mutants. Wild type and *tfc6* strains are the same as in Figures 2 and 3, and the B-box mutants are DDY5124 and DDY5126. IGV, integrative genomics viewer.

*PCL5* (*YHR071W*) is located on chrVIII, and its termination codon is approximately 150 base pairs upstream of the 3′-end of tDNA *tF(GAA)H1*, which is convergently transcribed. IGV profiles derived from the mutant strains (Figure 4A) suggested partial transcriptional readthrough past the normal *PCL5* terminator, resulting in 3′-extended mRNAs containing an additional approximately 400 bp on a fraction of the transcripts (red arrow). DESeq results were consistent with a 3′-UTR extension, showing an approximately 10-fold increase (padj =5.29E-06) of reads downstream of *PCL5* in the *tfc6* mutant strains. In order to validate this 3′-UTR extension of *PCL5*, we performed RT-PCR using internal and readthrough-specific primers on wild type, *tfc6* mutants, and again including two specifically constructed *tF(GAA)H1* B-box mutant strains. The results in Figure 4B confirmed the presence of *PCL5* readthrough, as extended transcripts were observed in both *tfc6*-under-expressing mutants and B-box mutants, but were not detected in the wild type strain.

#### De-repression (SPO74)

**Figure 5:**
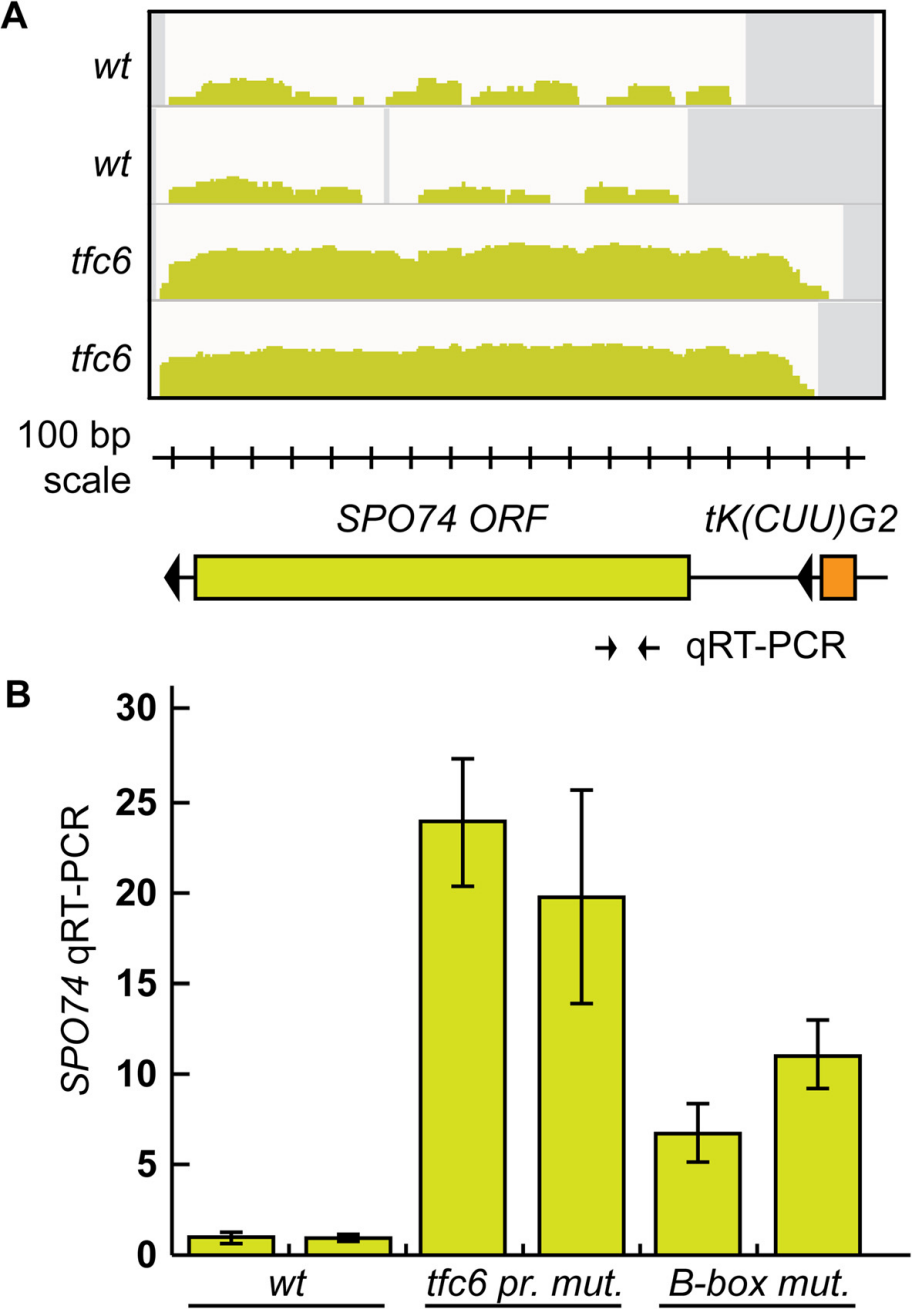
**De-repression of** ***SPO74*** **in** ***tfc6*** **and B-box mutants. A)** IGV profiles of *SPO74* showing apparent de-repression. Schematic diagram showing the relative position of the upstream tDNA, drawn to scale with the IGV profiles. The location of the qRT-PCR primers is shown below the *SPO74* ORF. **B)** Quantitative RT-PCR showing an increase in *SPO74* RNA in *tfc6* and B-box mutant strains relative to wild type. The B-box mutants were DDY5128 and DDY5129. IGV, integrative genomics viewer.

*SPO74* (*YGL170C*) is required for spore formation and is located on chrVII of *S. cerevisiae* [43]. As it is a sporulation specific gene, *SPO74* is not significantly transcribed in haploid or exponentially dividing *S. cerevisiae.* The tDNA *tK(CUU)G2* terminates approximately 300 bp upstream of the 5′-end of *SPO74*. Our mapped RNA-Seq reads and DESeq analysis suggested a moderate approximately 11-fold de-repression (padj =3.20E-25) of *SPO74* in the mutant strains compared to the low level of reads seen in wild type strains (Figure 5A). Quantitative RT-PCR (qRT-PCR) of *SPO74* mRNA levels was performed to confirm this apparent de-repression, and we again constructed strains containing targeted tDNA B-box mutations. Figure 5B shows the results of this analysis. The *tfc6* mutants showed an approximately 20-fold increase in transcripts within the *SPO74* coding sequence, and the B-box mutants expressed *SPO74* transcripts at a 7- to 10-fold higher level compared to wild type.

### Extra-transcriptional effects at non-tDNA, non-*ETC* loci bound by Pol III complexes

*S. cerevisiae* contain several non-tDNA Pol III transcribed loci, including the 5S rRNA gene *RDN5*, the U6 spliceosome RNA gene *SNR6*, snoRNA gene *SNR52*, RNase P RNA gene *RPR1*, signal recognition particle RNA gene *SCR1* and *RNA170*, a non-coding RNA of unknown function [1]. Additionally, under conditions of nucleosome depletion, transcription of *RNA170* is elevated, and transcription from the Pol III complex-bound *ZOD1* locus, which is not normally active under standard growth conditions, is de-repressed [42]. Inspection of RNA-Seq profiles at these loci demonstrates that in our *tfc6* mutants, adjacent Pol II transcription profiles were altered for all except *SNR52*. Effects at *RDN5* could not be included in the analysis since our RNA-Seq samples were depleted of ribosomal RNA.

**Figure 6:**
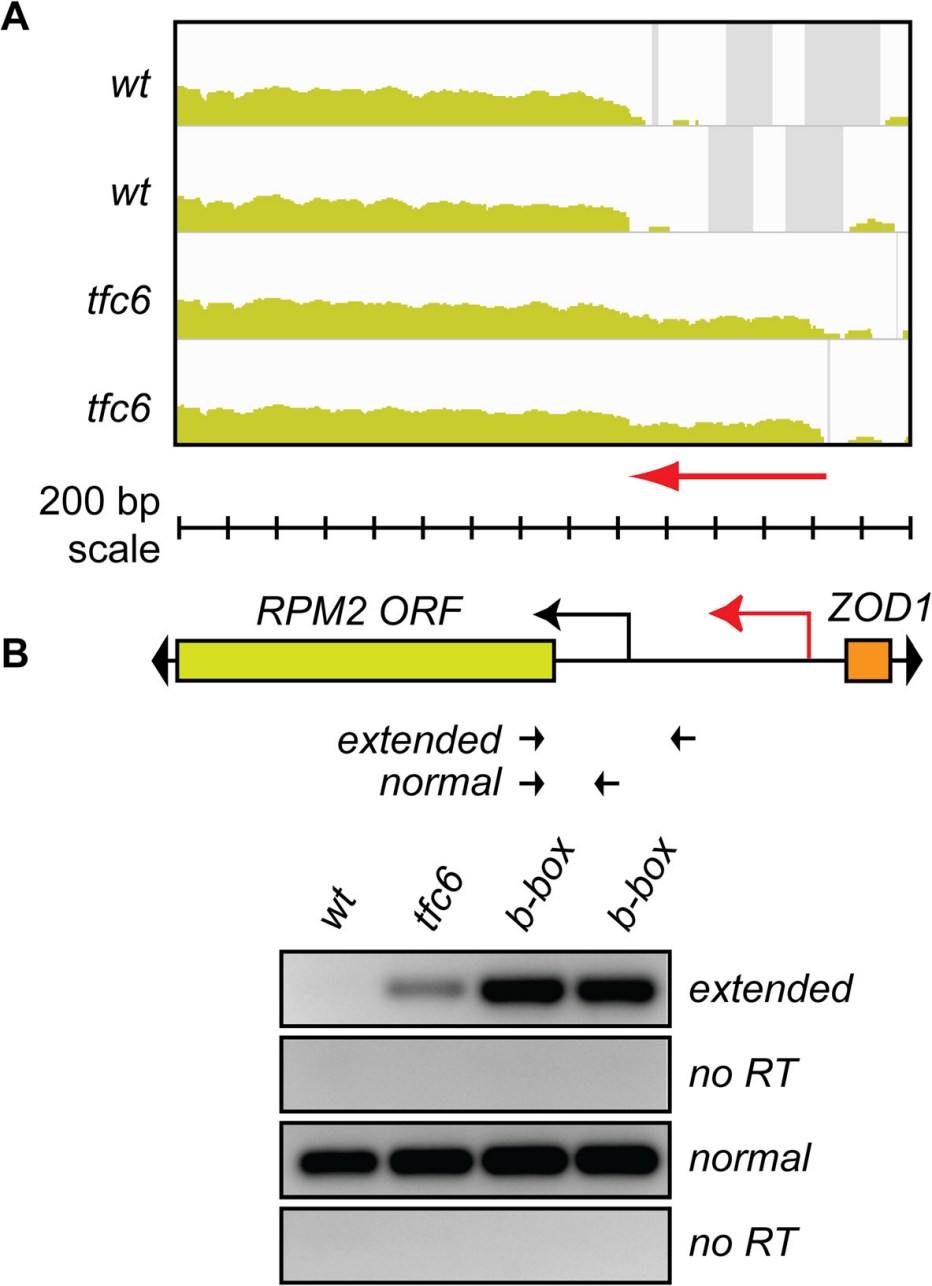
**5′-UTR extension at non-tDNA/non-ETC Pol III associated locus** ***ZOD1*** **-** ***RPM2*** **. A)** IGV profiles showing extended 5′-UTR of *RPM2* in *tfc6* mutants. **B)** Schematic of locus and location of primers. As before, extended transcripts are enriched in *tfc6* mutant and B-box mutant strains relative to wild type. Strains used were wild type DDY3630, *tfc6* DDY4300, and B-box mutants DDY5164 and DDY5165. IGV, integrative genomics viewer; Pol III, Polymerase III.

Figure 6 shows the effect of compromising Pol III complex assembly at the *ZOD1* locus. Here again, we observe an extended 5′-UTR in *RPM2* transcripts (red arrow in Figure 6A) in the *tfc6* mutants. These altered transcripts in one *tfc6* mutant were confirmed by RT-PCR, and are much more highly enriched in two strains containing specific mutations in the *ZOD1* B-box (Figure 6B). Although not confirmed by B-box mutagenesis, observed effects (determined by inspection of IGV profiles and DESeq analysis) at other non-tDNA loci in *tfc6* mutants include: *SNR6*, increased level of adjacent Ty1 LTR transcripts; *RPR1*, increased level of the adjacent *SUT088* intergenic transcript; and *SCR1*, increased level and 5′-extension of the adjacent uncharacterized gene *YER137C*. 5′-Extension of adjacent *RAD14* transcripts was confirmed in *tfc6* and *RNA170* B-box mutants (Q. Wang, unpublished data).

### 5′-Extended transcripts are compromised for translation

**Figure 7:**
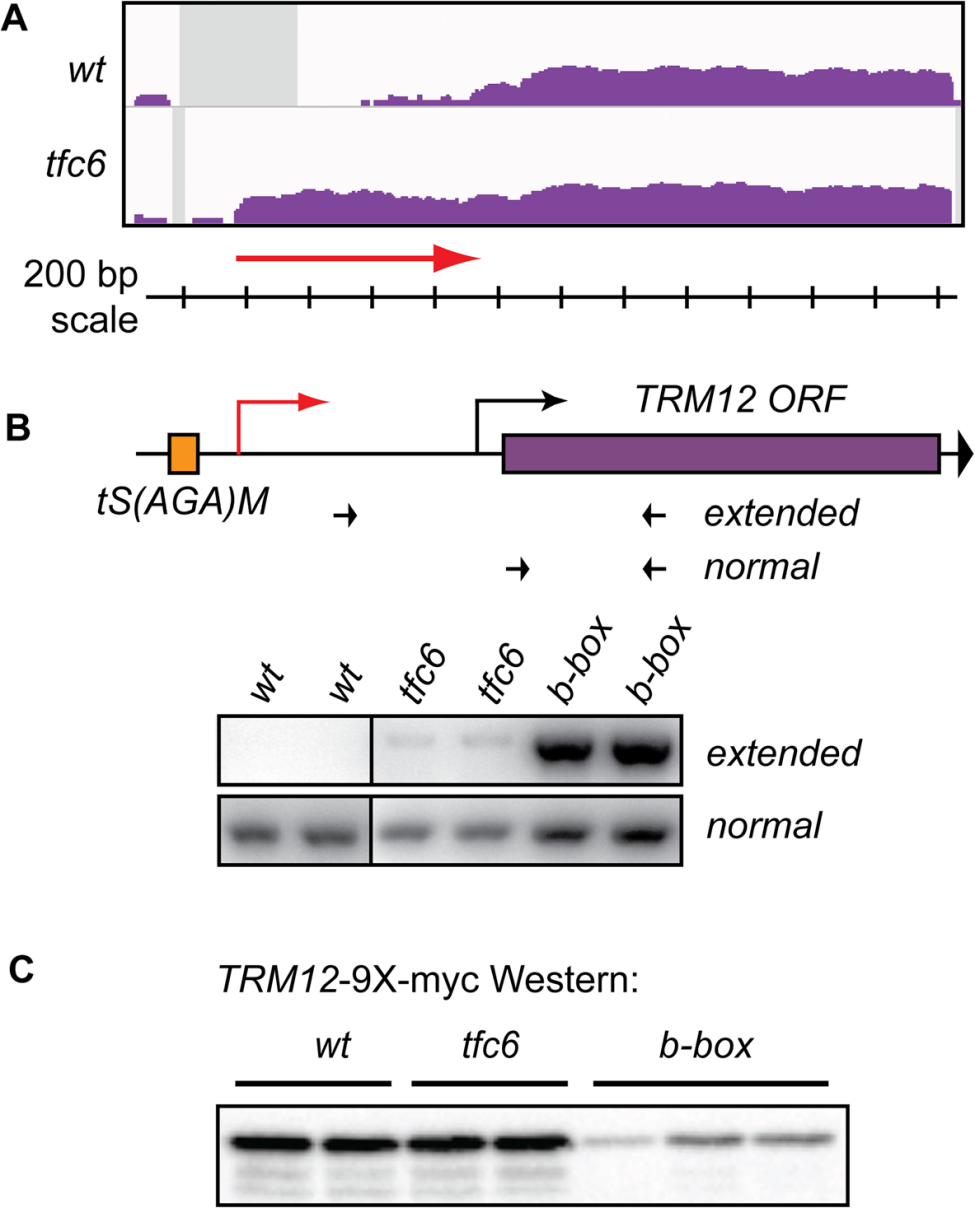
**5′-Extension of** ***TRM12*** **mRNA leads to reduced Trm12 protein levels. A)** IGV profiles showing apparent approximately 800 base extension of *TRM12* mRNA in *tfc6* mutant. **B)** Extended *TRM12* 5′-UTR levels are relatively low in *tfc6* mutants, but much more abundant in *tS(AGA)M* B-box mutants DDY5162 and DDY5163. Extended RNAs were detected by RT-PCR as in previous figures. **C)** Western blot of *TRM12*-9 × -myc strains shows reduced Trm12 protein levels in the B-box mutants. *TRM12*-9 × -myc tagged strains are: wild type, DDY5170 and DDY5171; *tfc6*, DDY5172 and DDY5173; and *ts(aga)m b-box*, DDY5174, DDY5205 and DDY5206. IGV, integrative genomics viewer.

Since we previously observed that 5′-extended *ATG31* transcripts created by *SUT467* readthrough were defective in translation of Atg31p [30], we tested protein expression of another gene that showed such a 5′-extension in this study. We chose *TRM12* (*YML005W*), as the IGV profile suggested that its mRNA might have a considerable extension of about 800 bases (Figure 7A, red arrow). Surprisingly, RT-PCR to detect this extended RNA showed only slight amplification in the *tfc6* mutant strains compared to wild type (Figure 7B), suggesting that a significant fraction of the DE intergenic reads at this locus might represent separate transcripts. However, in strains where the B-box of the upstream *tS(AGA)M* was mutated, we observed a much stronger signal in the RT-PCR analysis (Figure 7B), indicating that this 5′-extension is more prevalent when Pol III complex association is completely abolished.

We attached nine copies of the myc-epitope tag coding sequence to the end of *TRM12* in wild type, *tfc6* and B-box mutant strains. Western blot analysis of these strains using anti-myc antibody showed similar Trm12 protein levels in wild type and *tfc6* strains, but a significant reduction of Trm12p was observed in the B-box mutants (Figure 7C). These results, along with our previous analysis of *ATG31* protein levels demonstrate that, as expected, 5′extension of mRNAs created by disruption of adjacent Pol III complex formation impacts translation of the coding sequences.

## Discussion

The results presented here suggest that the presence or absence of DNA-bound Pol III complexes has a clear impact on neighboring chromosomal regions, as Pol II transcription start and termination sites near TFIIIC binding sites are altered in the *tfc6* and specific B-box mutant strains. We also confirm aspects of a previous microarray study in *S. cerevisiae* [31] demonstrating that numerous genes under the control of the transcription factor Gcn4p are up-regulated when RNA Pol III complex assembly is globally impaired (see Additional file 1: Table S5). This mis-regulation was not associated with proximity to the Pol III bound loci. That study, along with prior bioinformatics predictions of modest effects of tDNAs on neighboring Pol II promoters in yeast [22], suggested that global position effects of tDNAs on adjacent Pol II promoters, possibly including tgm silencing phenomena, were minimal. In contrast to those studies, we observe in Tfc6p depleted mutants numerous alterations of intergenic Pol II transcription that does occur in direct proximity to the compromised Pol III complex binding sites. We confirmed several of these globally induced effects by targeted mutation of the adjacent B-box to locally inhibit TFIIIC binding and Pol III complex formation. Since the effects are local, it is unlikely that formation of Pol III complexes is directly regulatory for the neighboring Pol II genes, but is a consequence of proximity.

A frequently observed effect in our Pol III mutants was extended 5′-UTRs of mRNAs adjacent to tDNAs, and as we have shown here for *TRM12*, and previously for *ATG31* [30] such 5′-extensions lead to significant inhibition of protein translation as would be expected. This raises an important issue in genomic analyses, as many studies report alterations in mRNA levels only, without assessing how mutation of DNA binding proteins, their binding sites, or perhaps even mutation of chromatin modifiers may affect TSS usage and, therefore, protein expression. Our data demonstrating that mutation of the *TFC6* promoter upstream of *ETC6* alters the TSS, and affects protein levels more severely than predicted by the modest decrease in mRNA levels (Figure 2), may implicate a similar scenario for other comparable mutations that inhibit the binding of proteins to DNA or chromatin.

As described in the introduction, compromised TFIIIC binding alters TSS integrity, and clearly inhibits Atg31 protein production [30], a phenotype that would likely have gone unnoticed had only mRNA levels been measured by qRT-PCR. Furthermore, globally compromised binding of the Pol III complex potentially affects translation of other mRNAs, as we observe numerous 5′-extended protein-coding transcripts. While such global effects have been alluded to previously [44], alterations in translational potential have largely been ignored in transcriptome studies. Recently, however, more attention is now being directed to this area in large-scale studies [45,46]. It is possible that a subset of phenotypes of gene expression mutants that were initially attributed to changes in mRNA levels might also involve effects on translation.

**Figure 8:**
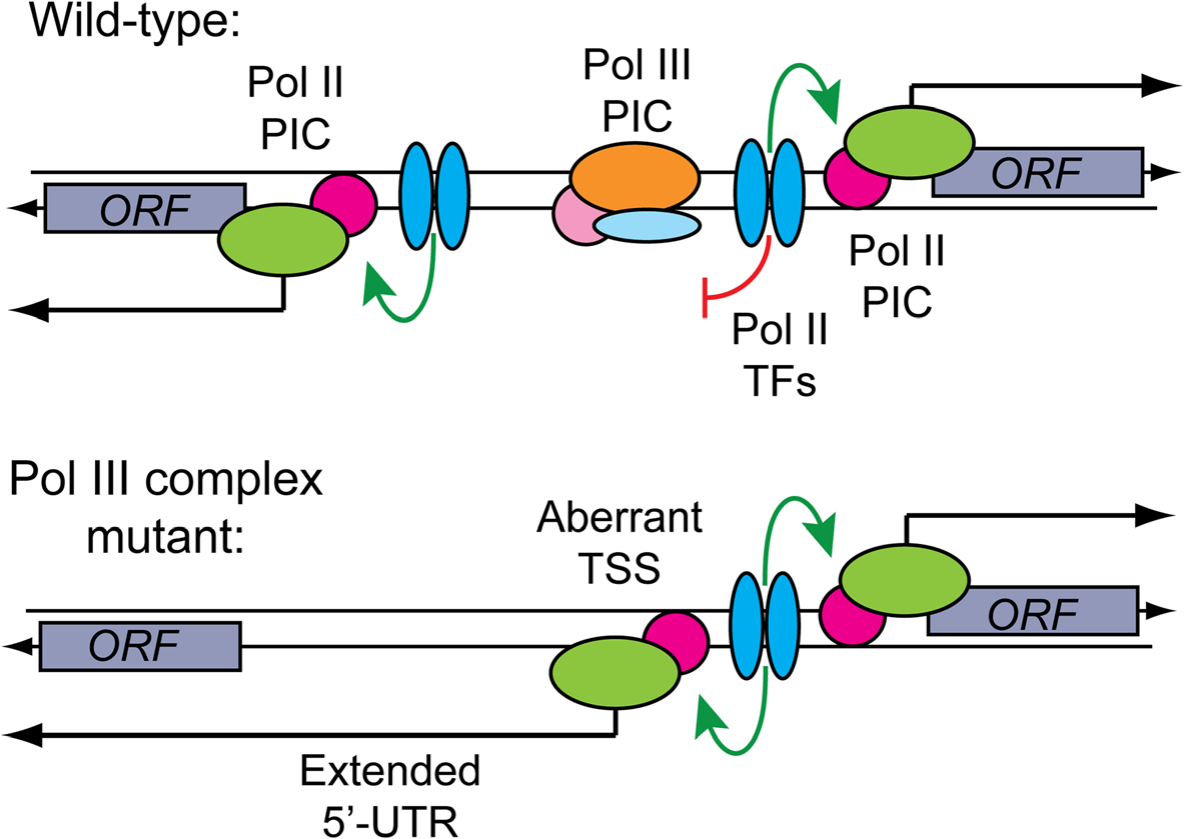
**Model for the appearance of 5′-extended and de-repressed Pol II transcripts in Pol III complex mutants.** In wild type cells, the bidirectional activity of transcription factor binding sites at some promoters is inhibited by the presence of a nearby fully or partially assembled Pol III complex (for example, *ETC* site). Compromised Pol III complex formation allows Pol II transcription factors to bidirectionally load Pol II preinitiation complexes (PIC). These events lead to the creation of aberrant transcription start sites (TSS), resulting in the extension of the 5′-UTR of the divergent gene. Upstream initiating Pol II may also inhibit normal PIC formation by transcriptional interference. This scenario may also be involved in the de-repression of *SPO74* (and other intergenic regions adjacent to tDNAs) observed when Pol III assembly at an upstream tDNA is compromised. In this case the aberrant transcript reads through chromatin-bound factors responsible for repression of *SPO74* in haploid cells. Pol II, polymerase II; Pol III, polymerase III.

Manual inspection and computational analysis of our RNA-Seq data at these loci reveal that the extended 5′-ends observed in *tfc6* mutants may normally be constrained by assembled Pol III complexes that prevent an upstream promoter from acting in a bidirectional manner. With the advent of tiling array and RNA-Seq technologies, the presence of pervasive and intergenic transcription in both prokaryotes and eukaryotes has been revealed. A significant fraction of such transcripts appears to arise from intrinsic bidirectional preinitiation complex formation directed by transcription factors bound upstream of active Pol II promoters [34,44]. Of the list of 5′-UTR extended loci in *tfc6* mutants given in Additional file 1: Table S6, the origin of many of the extensions is consistent with a model shown in Figure 8, which is based on the bidirectional model proposed by the Steinmetz lab. In these instances, it appears that one of a divergently transcribed gene pair is expressed at a high level, but in wild type cells, intrinsic bidirectional initiation is inhibited by the presence of adjacent chromatin bound Pol III complexes (Figure 8, upper panel). When Pol III complex formation is compromised, the bidirectional capacity of DNA bound transcription factors is enabled, allowing formation of a new intergenic Pol II TSS. Progression of Pol II from the new upstream TSS then leads to transcriptional interference of the normal divergent promoter, resulting in the extended 5′-UTRs (Figure 8, lower panel). Given that Pol III complexes exhibit both chromatin insulator and heterochromatin barriers [5], this bidirectional blocking activity could be considered as another type of ‘boundary’ element. Boundaries are defined as sequences that prevent regulatory elements from inappropriately affecting adjacent chromosomal regions; therefore, the blocking of bidirectional transcription by Pol III complexes can be viewed as insulating the divergent gene from the interfering effects of cryptic bidirectional transcription.

This mechanism also appears to be consistent with the observed de-repression of *SPO74*, as the *tK(CUU)G2* gene lies between *SPO74* and the divergently transcribed *SUA5* gene, the promoter of which appears to act bidirectionally in *tfc6* and tDNA mutants. We note here, however, that this de-repressed *SPO74* RNA is likely non-functional, as it begins far enough upstream to contain spurious translation starts and stops ahead of the actual *SPO74* AUG codon. Taken together with our previous work demonstrating extra-transcriptional functions of Pol III complexes (described in the introduction), we add to this list the ability to inhibit bidirectional pre-initiation complex recruitment associated with nearby Pol II transcription factor binding sites.

In addition to simply acting as a physical impediment to preinitiation complex formation, several potential mechanisms can be proposed for how Pol III complexes inhibit the bidirectionality of adjacent Pol II transcription factor binding sites. Several studies have demonstrated that Pol III complexes assembled at active tRNA genes strongly influence the positioning of neighboring nucleosomes in a dominant manner [16-18]. The loss of nucleosome phasing near tDNAs might uncover hidden cryptic TSS leading to the observed 5′-UTR extensions in our mutants. Nucleosome positioning at these loci might also involve the recruitment and activity of chromatin remodelers Isw1, Isw2 and/or RSC, loss of which has been shown to affect intergenic transcription and nucleosome positioning near tDNAs [16,47]. Sub-nuclear localization of genomic loci might also influence bidirectionality, as positioning of tDNAs to the nucleolus is required for tgm silencing by tDNAs [48], and most of the *ETC* sites in *S. cerevisiae* are known to localize to the nuclear periphery [49]. Regardless of the specific mechanism, it appears that assembled Pol III complexes, and possibly other DNA binding proteins, serve a secondary genomic function in maintaining the precision of Pol II TSS selection at some loci. Such functions may also provide an explanation for the evolutionary conservation of some of the non-transcribed *ETC* site regions in yeast [11].

The results presented here might also impact synthetic biology efforts to engineer the *S. cerevisiae* genome. In a pilot study that reported the construction of a minimal yeast chromosome III, eleven tDNAs were deleted without any significant effect on fitness [50]. Inspection of the IGV transcriptome profiles near all eleven chromosome III tDNAs in our *tfc6* mutants showed no significant effects on adjacent genes. A possible exception is at *SUP53*/*tL(CAA)C*, where apparent low level readthrough transcription from an upstream Ty2 element into the *LEU2* gene could potentially affect *LEU2* expression at the translational level. Interestingly, the chromosome III engineering project used a *leu2*Δ strain and functional *LEU2* as the marker gene for construction intermediates, so any deleterious readthrough effect at the native locus would not have been observed as leucine auxotrophy. Indeed, we observed compromised fitness upon mutation of the tDNA upstream of *ATG31*, as the mutant strains under-expressed Atg31p and were defective in autophagy induction [30]. It will be interesting to see whether extension of the minimal genome project to the remaining yeast chromosomes will reveal unforeseen fitness defects due to altered intergenic transcription upon deletion of specific tDNAs and other presumed non-essential sequences. The transcriptome data presented here might be predictive of a subset of such potential effects.

## Conclusions

Assembled RNA Pol III complexes are well documented to have significant extra-transcriptional effects on neighboring promoters. Using RNA-Seq analysis of Tfc6p under-expressing mutants, we identify the genome-wide impacts of Pol III complex binding sites, and show that numerous coding and intergenic transcripts were affected in the mutants. Many of the effects appear to be due to the release of bidirectional activity of neighboring promoters. Since we observe 5′-extended transcripts adjacent to the *tfc6* promoter mutation and at affected Pol III binding sites, it appears that alteration of TSSs may be a general result of altered protein-DNA binding within chromatin. The results may also be relevant to efforts to design a minimal yeast genome.

## Materials and methods

### Yeast strains and growth media

All yeast strains used in this study are derived from the W303-1a background, DDY2 (diploid strain, *MAT*α*/MAT***a***ade2-1/ADE2 his3-11/his3-11 leu2-3,112/leu2-3,112 LYS2/lys2*Δ*: trp1-1/trp1-1 ura3-1/ura3-1*) and DDY3 (haploid strain, *MAT***a***ADE2 his3-11 leu2-1,112 lys2*Δ*: trp1-1 ura3-1*). Genotypes of all yeast strains, descriptions of plasmids and a list of oligonucleotides used are listed in Additional file 4: Table S7. The *tfc6*-under-expressing yeast strains DDY4300 and DDY4301 containing a 12-bp mutation in the *TFC6* promoter were described previously [32]. For specific tDNA B-box mutant yeast strains, the entire B-box sequence was scrambled by standard oligonucleotide-mediated site-directed mutagenesis. For *ETC* site mutant yeast strains, the conserved cytosine residue within the B-box was mutated to guanine. All mutations were verified by Sanger sequencing. All B-box mutants were integrated by standard yeast genetic techniques, involving deletion of the tDNA or *ETC* site by replacement with *URA3*, followed by transformation of site-directed mutagenized fragments and selection on 5-fluoroorotic acid (5-FOA) media. Each integrated mutation was verified by PCR and either sequencing or detection of an inserted *Drd* I restriction site inserted in place of the B-box sequence. 9 × -myc-tagged strains used for Western Blot analysis containing an integrated 9 × -myc-*TRP1* cassette were created as described [51], and were verified by PCR and sequencing of the junction of the gene through the entire epitope tag coding sequence before Western blot analysis.

### RNA extraction and RNA-Seq library preparation

Total RNA was extracted using a minor modification of a standard phenol/chloroform protocol as described [52]. Each strain was grown in rich YPD media (1% yeast extract, 2% peptone, 2% dextrose) from an initial A_600_ of 0.15 to mid log phase (A_600_ = 1.0) before harvesting cells for RNA preparation. Residual genomic DNA was removed by treatment with RQ1 DNase (Promega Madison, WI, USA M6101) according to the manufacturer’s protocol. Prior to Illumina library preparation, ribosomal RNA was depleted using RiboZero (Yeast) Kit (Epicentre/Illumina San Diego, CA, USA). RNA-Seq libraries were prepared with the Illumina TruSeq stranded RNA sample prep kit, which results in 5′- to 3′-strand-specific libraries. The four barcoded libraries were pooled and quantitated by qPCR, and the pool was sequenced for 101 cycles on one lane of a HiSeq2000, using a TruSeq SBS sequencing kit version 3 and processed with Casava 1.8.2, following the manufacturer’s instructions (Illumina, San Diego, CA). Library preparation and sequencing was performed by the Roy J. Carver Biotechnology Center at the University of Illinois at Champaign-Urbana.

### Quality check and read mapping

A total of approximately 196 million 100-bp reads were generated for the four RNA-Seq libraries. The quality-filtered reads from the Casava pipeline were further assessed with FASTQC software [53]. Across all four samples, an average Phred Score of greater than 32 was reached at each base position. We mapped reads against the W303 reference genome (from the *Saccharomyces* Genome Database, 2012 version, [54]) for each sample by using Bowtie2 [55] with the preset option for highest accuracy and sensitivity. Only uniquely mapped reads were considered for further analysis. Custom scripts written in Perl and Python were used together with Samtools [56] and Bedtools [57] to process and organize data files for downstream analysis. IGV was used to qualitatively assess and visualize the mapped reads to the reference genome [58]. Strand-specific bedgraph files of each sample were imported into IGV along with the W303_ALAV0000000.gff file as the reference.

### Differential gene expression level analysis

Differential expression of biological replicates between wild type and mutant samples was determined using DESeq [59], based on the number of RNA-Seq reads uniquely mapped to defined genomic loci and regions. As we were also interested in 5′-UTR or 3′-UTR extensions of transcripts, we separated known annotated protein coding sequences from the entire W303 genome and designated the remainder as intergenic regions, with Watson and Crick strands also separated. Intergenic regions longer than 500 bps were demarcated into 500-bp sized windows. Differential expression analysis was performed on coding sequence and intergenic regions separately by fitting uniquely mapped read counts to negative binomial distribution. Significantly differentially expressed transcripts were detected based on the padj cutoff value 0.05.

### RT-PCR and quantitative RT-PCR

First strand cDNA was synthesized from 500 ng total RNA after DNAse treatment (RQ1 DNAse, Promega M6101), using the ProtoScript First Strand cDNA Synthesis Kit (New England Biolabs Ipswich, MA, USA #E6300S). Gene-specific and/or random primed cDNA was made to confirm altered mRNA levels and extended transcripts for selected differentially expressed loci. Primers were designed for each locus to detect altered and normal transcripts in wild type and mutant strains (relative locations are marked in each figure; all oligonucleotide sequences are listed in Additional file 4: Table S7). Quantitative reverse transcription PCR (qRT-PCR) was performed as 25 μl reactions with 1:4 diluted cDNA, and primers were diluted to a final concentration of 0.5 μM. Sybr Green super mix (Bio-Rad Hercules, CA, USA 170-8882) was added and reactions were run and analyzed on a Bio-Rad MyiQ with 60°C annealing temperature. Results were normalized to *ACT1* mRNA amplified by primers DDO402-403 and graphed as fold-change between wild type and mutant strains.

### Protein extraction and western blot

Myc-tagged strains were grown in 40 ml YPD culture at 30°C with shaking until the O.D. of A_600_ reached approximately 0.8 to 1.0. Cells were collected and washed in 1 ml ice-cold 1XTBS/0.05% NaN_3_/50 mM NaF. Cell pellets were resuspended in 100 μl of lysis buffer (20 mM Tris-HCL, 125 mM KOAc, 4 mM MgCl_2_, 0.5 mM ethylenediaminetetraacetic acid (EDTA), 5 mM sodium bisulfite, 0.1% Tween-20, 12.5% glycerol, 1 mM dithiothreitol (DTT), 2 μg/ml leupeptin, 2 μg/ml pepstatin A, 1 mM phenylmethylsulfonyl fluoride (PMSF)) plus an equal volume of acid washed 0.5 mm glass beads, and disrupted in a Biospec Mini-beadbeater, 5 × 15-second pulses at 4°C. Cell lysates were cleared by centrifugation at 20,000 × g for 15 minutes in a refrigerated microfuge at 4°C, protein concentration of the supernatant was measured using standard Bradford assay (Bio-Rad), and 60 μg of total protein per lane was loaded on an 8% acrylamide SDS-PAGE gel. Proteins were transferred to Immobilon membrane (EMD Millipore, Billerica, MA, USA), with Blotto for one hour at room temperature, and then primary anti-Myc antibody (9E10, Santa Cruz Biotechnology, Santa Cruz, CA, USA) was added. After overnight incubation and washing of blots, anti-mouse Ig-horseradish peroxidase secondary antibody (GE healthcare NXA931 Piscataway, NJ, USA) was added in Blotto for two hours at room temperature. Immuno-star Western chemiluminescent kit (Bio-Rad 170-5070) was used for detection. Signals were captured and analyzed by using a ChemiDoc XRS + systems with Image Lab software (Bio-Rad).

### Data access

The raw transcriptome sequencing data has been deposited to the NCBI Sequence Read Archive (SRA) Database, Submission ID: SUB647823, BioProject ID: PRJNA258413. The accession number is [SRP045581].

## Additional files

Additional file 1: Table S1. Transcriptome read data; **Table S2.** Differentially expressed open reading frames and intergenic regions; **Table S5.** Comparison of results to Conesa *et al*. [31] study; **Table S6.** List of statistically significant upregulated intergenic regions in the *tfc6* mutants; **Figure S1.** Scatter plots for separate DESeq analyses; and **Figure S2.** RNA-Seq confirmation of readthrough transcription previously identified at *ATG31* in the *tfc6* promoter mutant. Additional file 2: Table S3. DESeq results of annotated open reading frame sequences comparing wild type and tfc6 mutants. Additional file 3: Table S4. DESeq results of intergenic region sequences comparing wild type and tfc6 mutants. Additional file 4: Table S7. Sheet 1, genotypes of all yeast strains constructed and used in this study; Sheet 2, list of all plasmids constructed and used in this study; Sheet 3, list of all oligonucleotides used in this study.
