# Supplementary material for: Compromised RNA polymerase III complex assembly leads to local alterations of intergenic RNA polymerase II transcription in Saccharomyces cerevisiae
Source: BMC Biol. 2014 Oct 28;12:89. doi: 10.1186/s12915-014-0089-x (PMC4228148; doi:10.1186/s12915-014-0089-x)

***Compromised RNA Polymerase III complex assembly leads to local alterations of intergenic RNA Polymerase II transcription in Saccharomyces cerevisiae.***

**Qing Wang, Chance M. Nowak, Asawari Korde, Dong-Ha Oh, Maheshi Dassanayake, and David Donze**

**Supplementary tables and figures.**

**Table S1**. Read data for RNA-seq samples of the four yeast strains analyzed in this study. The percentage of total reads mapped to the yeast genome were: DDY3, 96.53%; DDY3630, 97.09%; DDY4300, 95.85%; and DDY4301, 95.55%.





**Table S2.** Characterization of Differentially Expressed (DE) ORFs and intergenic regions with respect to proximity to potential Gcn4p and TFIIIC binding sites. Factor binding sites were inferred from the *Saccharomyces* Genome Database Genome Browser tracks. None or Others refer to loci that are not adjacent to documented Gcn4p or TFIIIC binding sites. This data was used to construct figures 1A and 1C.


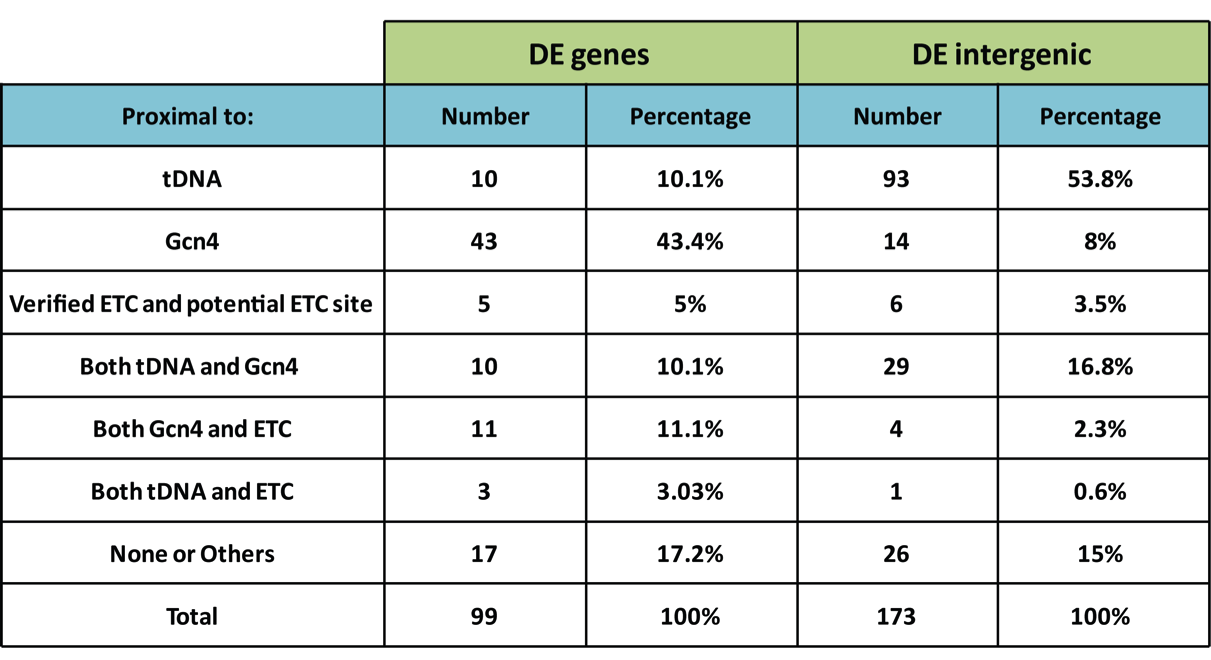


**Table S3.** DESeq results of all annotated open reading frames in the yeast genome, available in supplementary information as separate Excel file.

**Table S4.** DESeq results of all intergenic regions with identified expression units in the yeast genome, available in supplementary information as separate Excel file.

**Table S5.** Comparison of the top mis-regulated ORFs from Conesa et al. 2005 (using TFIIIC, TFIIIB, and RNA Polymerase III subunit mutants) with the RNA-seq analysis of Tfc6p under-expressing mutants. Results are similar with the exception of *BAT1*, *CTF13* and *MTG1*, which are not up-regulated in the *tfc6* mutant when analyzed by strand-specific RNA-seq. The discrepancy with two of these genes appears to be due to overlapping convergent transcription of *CTF13* with *SNO1* and *MTG1* with *SNZ1,* which likely led to increased *CTF13* and *MTG1* signal artifacts on the ORF microarray. Numbers in parentheses indicate DESeq analyses where the padj value was >0.05.


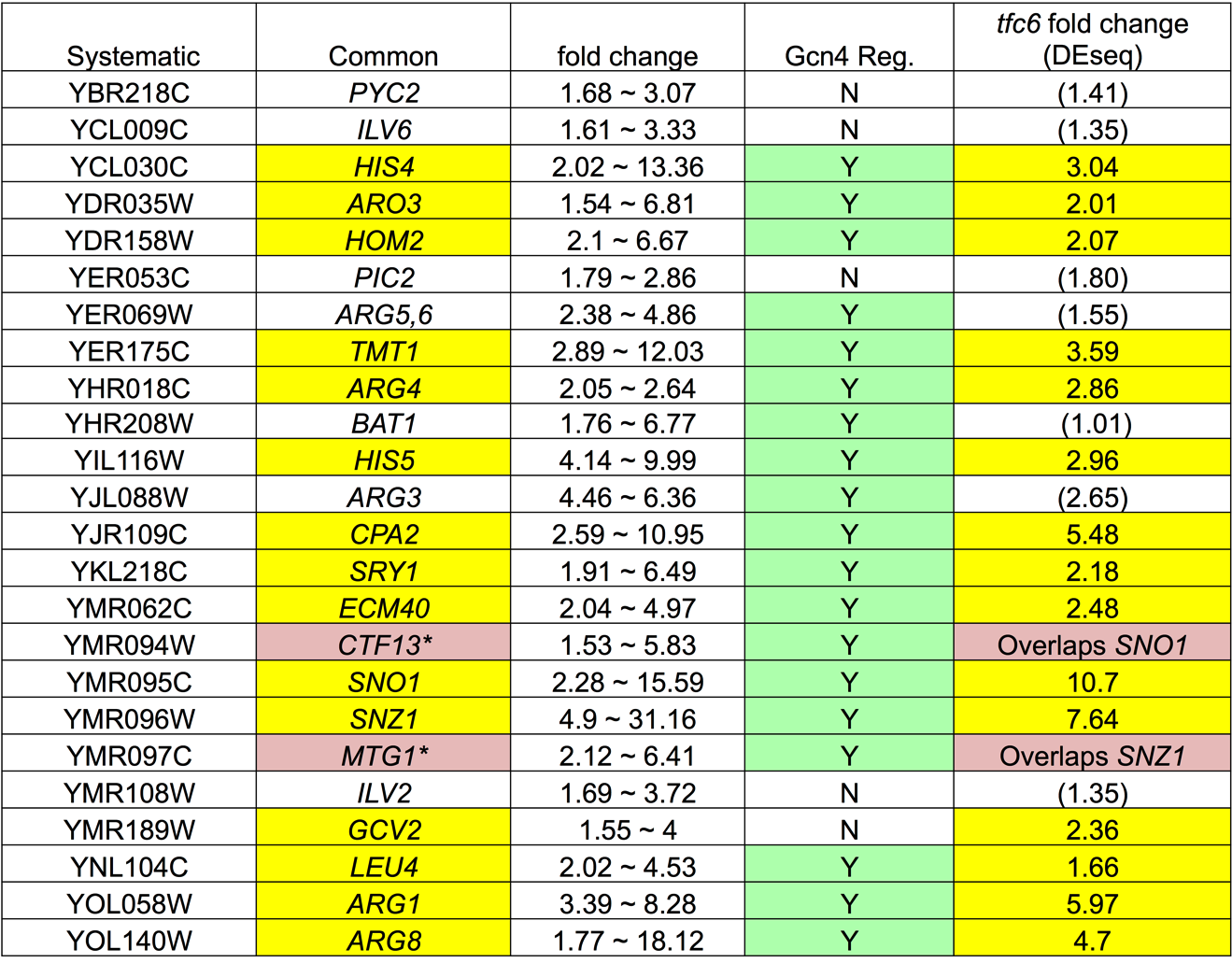


**Table S6.** List of statistically significant upregulated intergenic regions in the *tfc6* mutants, and their proximity to other genomic features (Y=yes, N=no). Approximate base pair distances to tDNAs and Gcn4 sites are given in table S4.


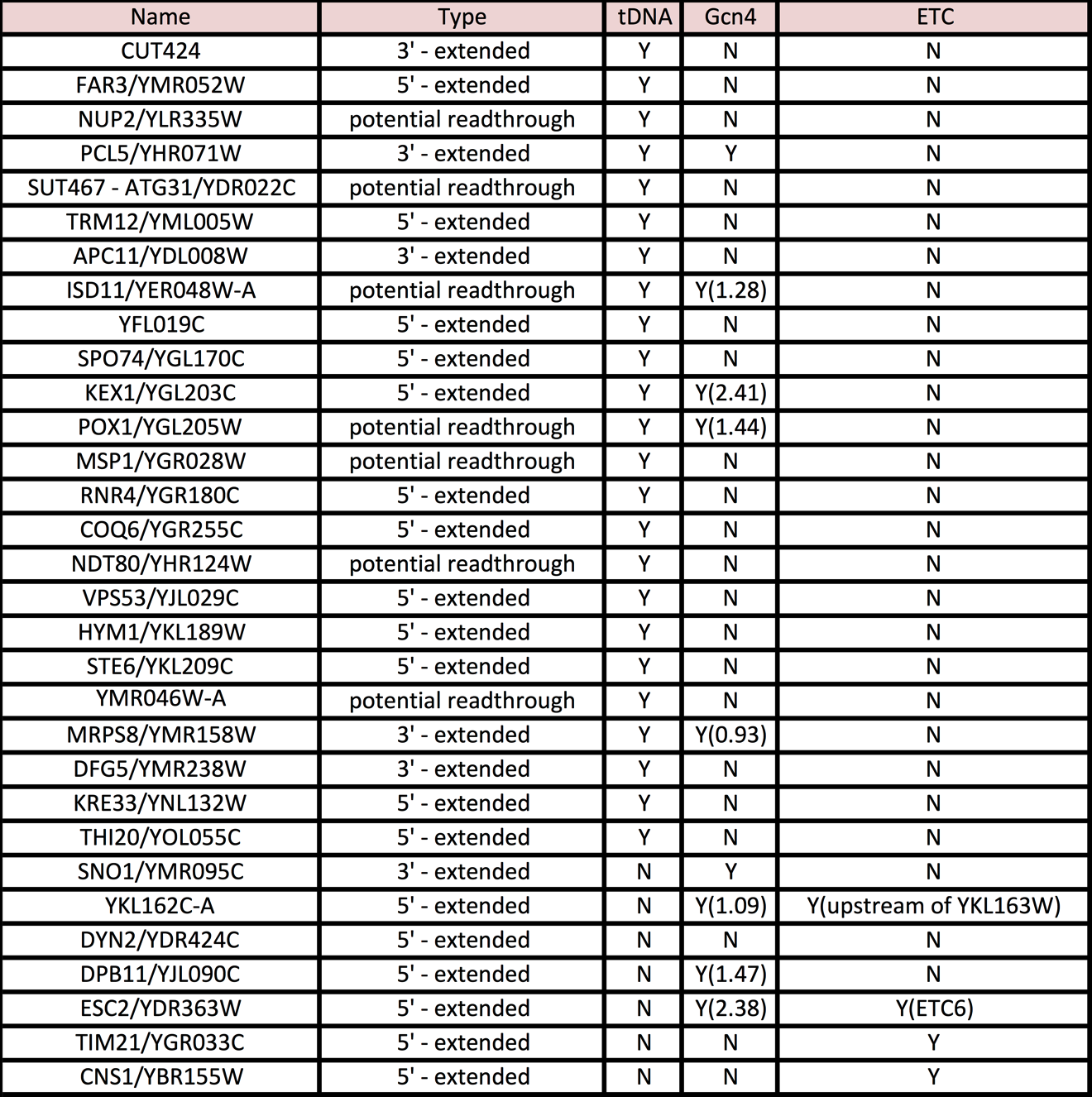


**Table S7**. Yeast strains, plasmids, and oligonucleotides used, available as a separate Excel file in the Supplementary Information.

**Figure S1.** Scatter plots for separate DESeq analyses of open reading frames (A), and intergenic regions (B). The X-axis represents the averaged normalized RNA-Seq count for wild type and mutant transcript models and the Y-axis shows the corresponding log fold change of transcript intensity between mutants and wild-type samples. Red dots represent statistically significant differentially expressed regions compared to all differentially expressed regions shown by black dots. The padj cut off value was set to < 0.05.

A
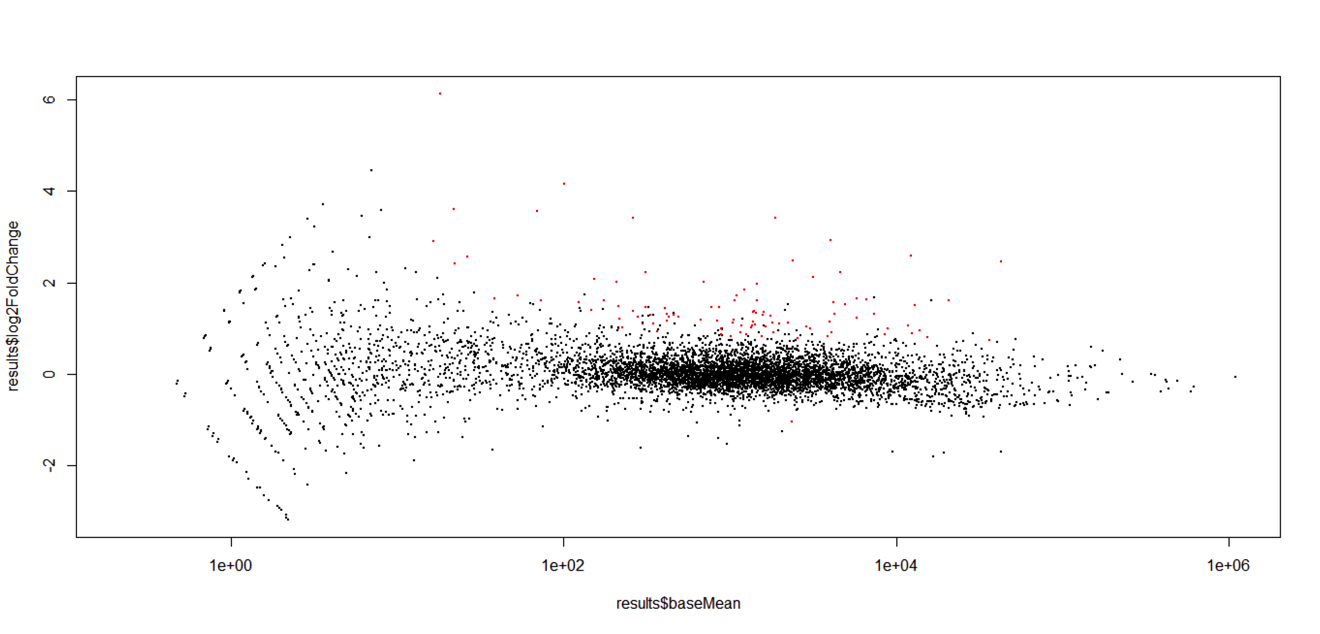


B
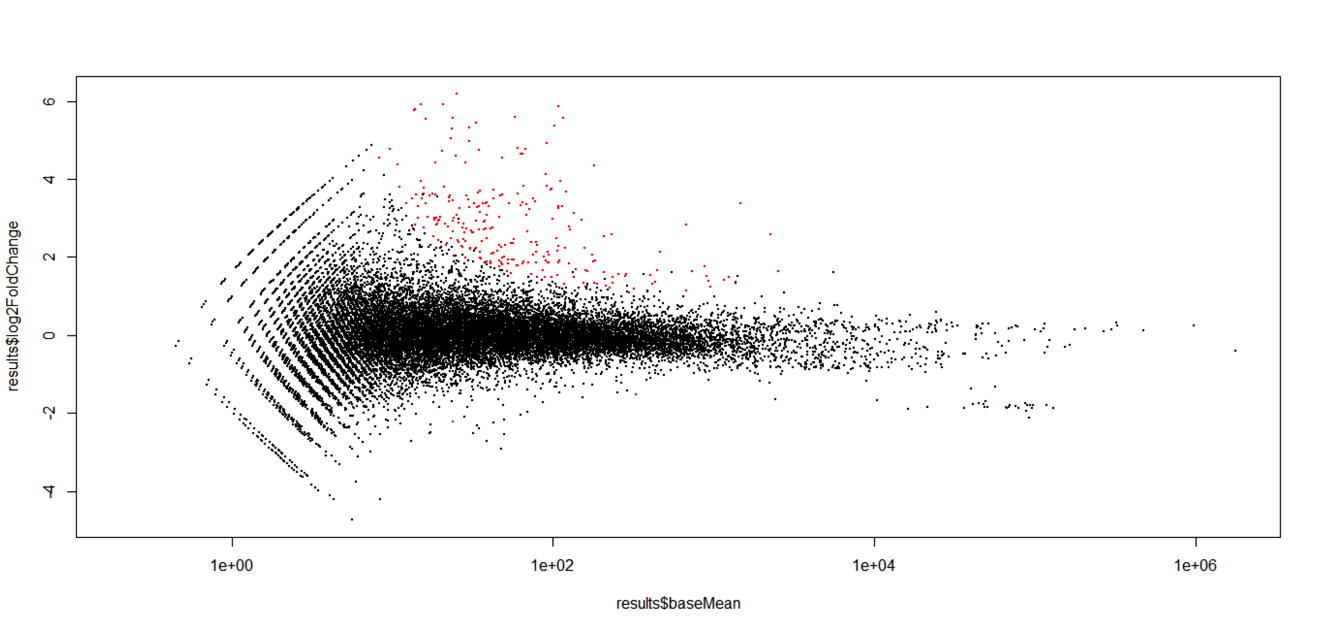


**Figure S2.** RNA-Seq identifies previously characterized readthrough of *SUT467* into *ATG31* in *tfc6* mutants. Transcriptome reads in the region of the tDNA are minimal in wild type strains, and are enriched in the mutants, consistent with the presence of extended *ATG31* transcripts identified by Northern blotting and RT-PCR in Korde *et al*. (2014). DESeq analysis determined a 2.4-fold increase in the readthrough region in mutants over wild type, padj = 0.036 (Table S4).


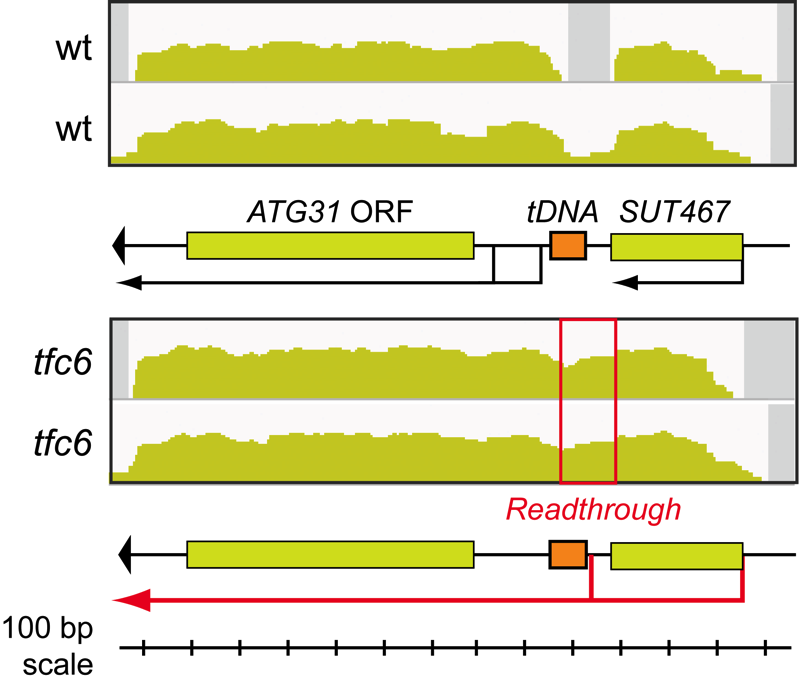

Supplement: Additional file 1: Table S1. — Transcriptome read data; Table S2. Differentially expressed open reading frames and intergenic regions; Table S5. Comparison of results to Conesa et al. [31] study; Table S6. List of statistically significant upregulated intergenic regions in the tfc6 mutants; Figure S1. Scatter plots for separate DESeq analyses; and Figure S2. RNA-Seq confirmation of readthrough transcription previously identified at ATG31 in the tfc6 promoter mutant. [file 12915_2014_89_MOESM1_ESM.docx]
